# RSM Modeling and Optimization of CO_2_ Separation from High CO_2_ Feed Concentration over Functionalized Membrane

**DOI:** 10.3390/polym14071371

**Published:** 2022-03-28

**Authors:** Nadia Hartini Suhaimi, Yin Fong Yeong, Norwahyu Jusoh, Thiam Leng Chew, Mohammad Azmi Bustam, Muhammad Mubashir

**Affiliations:** 1 Department of Chemical Engineering, Universiti Teknologi PETRONAS, Bandar Seri Iskandar 32610, Malaysia; nadia_17005943@utp.edu.my (N.H.S.); norwahyu.jusoh@utp.edu.my (N.J.); thiamleng.chew@utp.edu.my (T.L.C.); azmibustam@utp.edu.my (M.A.B.); 2 CO_2_ Research Centre (CO_2_RES), R&D Building, Universiti Teknologi PETRONAS, Bandar Seri Iskandar 32610, Malaysia; 3 Centre of Research in Ionic Liquids (CORIL), Universiti Teknologi PETRONAS, Bandar Seri Iskandar 32610, Malaysia; 4 Department of Petroleum Engineering, Faculty of Computing, Engineering & Technology, School of Engineering, Asia Pacific University of Technology, and Innovation, Kuala Lumpur 57000, Malaysia; mubashir@apu.edu.my

**Keywords:** functionalized MOFs, high CO_2_ concentration, optimization, RSM

## Abstract

The challenges in developing high CO_2_ gas fields are governed by several factors such as reservoir condition, feed gas composition, operational pressure and temperature, and selection of appropriate technologies for bulk CO_2_ separation. Thus, in this work, we report an optimization study on the separation of CO_2_ from CH_4_ at high CO_2_ feed concentration over a functionalized mixed matrix membrane using a statistical tool, response surface methodology (RSM) statistical coupled with central composite design (CCD). The functionalized mixed matrix membrane containing NH_2_-MIL-125 (Ti) and 6FDA-durene, fabricated in our previous study, was used to perform the separation performance under three operational parameters, namely, feed pressure, temperature, and CO_2_ feed concentration, ranging from 3.5–12.5 bar, 30.0–50.0 °C and 15–70 mol%, respectively. The CO_2_ permeability and CO_2_/CH_4_ separation factor obtained from the experimental work were varied from 293.2–794.4 Barrer and 5.3–13.0, respectively. In addition, the optimum operational parameters were found at a feed pressure of 12.5 bar, a temperature of 34.7 °C, and a CO_2_ feed concentration of 70 mol%, which yielded the highest CO_2_ permeability of 609.3 Barrer and a CO_2_/CH_4_ separation factor of 11.6. The average errors between the experimental data and data predicted by the model for CO_2_ permeability and CO_2_/CH_4_ separation factor were 5.1% and 3.3%, respectively, confirming the validity of the proposed model. Overall, the findings of this work provide insights into the future utilization of NH_2_-MIL-125 (Ti)/6FDA-based mixed matrix membranes in real natural gas purification applications.

## 1. Introduction

The East Natuna Block, located in the Greater Sarawak Basin near Natuna Island, Indonesia, is the largest natural gas field in Southeast Asia. It contains 46 trillion cubic feet (Tcf) of natural gas, and is identified as a high carbon dioxide (CO_2_) content gas field, with CO_2_ concentrations up to 70 mol%. In addition, about 13 trillion cubic feet (Tcf) of high CO_2_ gas fields in Malaysia remain unexploited, with CO_2_ concentrations of more than 40 mol%. According to the requirements of the gas processing plant, the CO_2_ content should be reduced to 8 mol% in order to comply with the criteria of the downstream sales gas process [1]. High CO_2_ contents can reduce the heating value of natural gas [2] and cause corrosion issues in pipelines in the presence of water [3]. Thus, it is crucial to investigate technologies for separating CO_2_ from methane at high CO_2_ feed concentrations. Among conventional technologies such as absorption [4], adsorption [5], cryogenic distillation, and membrane separation [6], membrane separation has gained the most attention due to its small footprint, simple operation and energy efficiency [7].

The evolution of membrane materials in separation technology started with polymeric, followed by inorganic, mixed matrix and metal organic frameworks (MOFs), etc. Mixed matrix membranes comprised of polymeric material and inorganic filler were developed to address the trade-off in performance experienced by polymeric membranes [8] and reproducibility concerns regarding the fabrication of inorganic membranes [9]. Lately, incorporating MOF fillers in polymeric membranes for the formation of mixed matrix membranes has been the focus of many researchers due to the fact that the high porosity of MOF fillers can improve gas separation performance [10].

On top of that, numerous efforts have been made by researchers to further enhance the separation performance of mixed matrix membranes by functionalizing metal-organic frameworks (MOFs) fillers with -NH_2_ functional groups. The presence of -NH_2_ in the filler induces strong affinity toward CO_2_ via hydrogen bonding, which enhances the interaction between CO_2_ and the filler framework [11,12], and subsequently, improves the performance of the membrane in terms of CO_2_ separation. Owing to its tunable pore size and affinity to CO_2_ gas [13,14,15], NH_2_-MIL-125 (Ti) has recently been studied as a filler for the formation of membranes for gas separation [3,16,17]. Previous studies reported that the separation performance of NH_2_-MIL-125 (Ti)/polysulfone mixed matrix membranes was superior to that of neat polysulfone membranes [17]. This finding was attributed to the existence of porous fillers, which created an extra pathway for the penetration of CO_2_ gas and promoted the diffusion of gas molecules through the membrane. Furthermore, in another work, a NH_2_-MIL-125 (Ti)/Matrimid mixed matrix membrane was fabricated for CO_2_/CH_4_ separation and a CO_2_/CH_4_ gas pair selectivity of 50 was obtained when 15 wt% filler was loaded into the membrane [16]. This was mainly due to the small aperture size (6 Å) of NH_2_-MIL-125 (Ti), which allowed CO_2_ molecules to interact with the -OH groups in Ti clusters, as well as interacting with -NH_2_ groups on the linker. Also, our previous study discovered that the inclusion of NH_2_-MIL-125 (Ti) filler in 6FDA-durene polymer matrices significantly improved the CO_2_ permeability and CO_2_/CH_4_ gas pair selectivity, i.e., by 119% and 331%, respectively, compared to neat 6FDA-durene membranes [3]. The improvement in performance was mainly attributed to the high porosity of NH_2_-MIL-125 (Ti) fillers and the attraction of amine functional groups to CO_2_ molecules. Although several studies have been published on the utilization of functionalized MIL-125 (Ti) as a filler for the preparation of mixed matrix membranes in CO_2_/CH_4_ separation, these studies mostly focused on single gas permeation, binary gas separation (50:50) and the effect of feed pressure on the membrane performance [16,17]. Hence, further research evaluating the separation performance of the membrane at high CO_2_ feed concentrations and various temperatures and CO_2_ feed pressures in binary gas mixtures is still crucial.

According to the findings in the literature, operating temperature influences the CO_2_ permeability of the membrane by affecting the mobility of the polymer chain and free volume [18] in the polymer, thus resulted in a change of CO_2_ diffusivity [19]. In contrast, feed pressure may affect the gas solubility in the membrane, which affects the CO_2_ permeability [11]. Furthermore, the CO_2_ concentration in the feed can cause significant changes in CO_2_ permeability through the membrane, which is mainly due to the higher sorption capability of CO_2_ compared to CH_4_. Hence, study of the correlation between the operational parameters and membrane separation performance is essential. In fact, several approaches, including response surface methodology (RSM), full factorial design and Taguchi methods, are available to investigate these questions.

For an experimental assessment and the optimization of the separation parameters, including significant responses and independent variables, comprehensive analytical tools and efficient optimization tools are practical. The multivariant method enabled the identification of the interaction between the variable parameters and responses. Furthermore, for our optimization study, the response surface methodology (RSM) was identified as the optimal tool. This technique combines the mathematical and statistical analyses by reducing the number of experiments. As a result, various researchers have reported the application of the RSM method in their studies.

Jusoh et al. [20] investigated the effect of pressure, temperature and CO_2_ feed concentration on CO_2_/CH_4_ binary gas separation over a ZIF-8/6FDA-based mixed matrix membrane. They reported that the optimum parameter for CO_2_/CH_4_ separation performance was obtained at a feed pressure of 4.76 bar, a temperature of 30 °C, and a CO_2_ feed concentration of 90 mol%. The latest study reported by Afarani et al. [18] utilized a polyurethane–zeolite 3A mixed matrix membrane to assess the impact of three independent variables, namely, zeolite content (0–24 wt%), operating temperature (25–45 °C), and operating pressure (0.2–0.1 MPa), on the single gas permeation performance. The optimum gas permeation performance of polyurethane–3A zeolite membrane was identified at a zeolite loading of 18 wt%, a temperature of 30 °C, and a pressure of 0.8 MPa. It can be observed that the RSM method has great potential for analyses of the relationship between the operational parameters and responses. Thus, the process of optimization of the separation performance over NH_2_-MIL-125 (Ti)/6FDA-durene mixed matrix membranes by varying the operational parameters including feed pressure, temperature and CO_2_ feed concentration using RSM method is presented in this study.

The 7.0 wt% NH_2_-MIL-125 (Ti)/6FDA-durene mixed matrix membrane reported in our previous work [3] was used in this study, since the membrane demonstrated the best performance in CO_2_ and CH_4_ single gas permeation testing. First, the interaction of operational parameters, including feed pressure (A), temperature (B), and CO_2_ feed concentration (C), toward the CO_2_ and CH_4_ permeability and CO_2_/CH_4_ separation factor were analyzed in detail using the RSM statistical method paired with the CCD optimization tool. Then, the significance of models was further evaluated by using analysis of variance (ANOVA). Next, 3D surface plots were used to visualize the linear, quadratic, and interaction between the operating parameters and significant responses. Subsequently, the optimum condition for the separation performance was generated and validated by experimental work.

## 2. Materials and Methods

### 2.1. Materials

Synthesis of NH_2_-MIL-125 (Ti) fillers required trimethylacetic acid (99%), N,N-dimethylformamide (DMF), 2-aminoterephthalic acid (98%), terephthalic acid (98%), acetonitrile (99.8%) and tetraisopropyl ortthotitanate (97%). These chemicals were purchased from Sigma Aldrich (Missouri, MO, USA) and were used without further purification.

Synthesis of 6FDA-durene polyimide required two monomers, i.e., durene diamine (99%) and 6FDA dianhydride (99%). Durene diamine was purified through recrystallization using methanol, while 6FDA-dianhydride was purified via the vacuum sublimation technique. Vacuum distillation was used to purify N-methyl-2-pyrrolidone (NMP). Propionic anhydride (≥98%), triethylamine (≥99%), methanol (≥99.9%) and dichloromethane (≥99.8%) were purchased from Merck (Massachusetts, MA, USA) and were used as received.

The premixed gases used for the gas separation test were purchased from Air Products Sdn Bhd, (Kuala Lumpur, KL, Malaysia) with CO_2_ compositions ranging from 15 mol% to 70 mol%.

### 2.2. Synthesis of 6FDA-Durene

In this study, 6FDA-durene polyimide was synthesized using a dual-step procedure reported in the literature [21]. First, equimolar amounts of durene diamine and 6FDA dianhydride monomers were dissolved in NMP for 24 h at room temperature under a nitrogen atmosphere. Next, polyimide was formed after adding propionic anhydride and triethylamine to the mixture solution. Then, the solution was precipitated in methanol and washed several times with methanol. The resulting polymer was dried under a vacuum for 24 h at 150 °C.

### 2.3. Synthesis of NH_2_-MIL-125(Ti)

The NH_2_-MIL-125 (Ti) filler was synthesized by following the method reported in our previous work [3]. Firstly, 17.5 g of pivalic acid was dissolved in a mixture solution containing 125 mL of acetonitrile and 5 mL of tetraisopropyl orthotitanate. Next, the mixture solution was placed in a Teflon-lined autoclave reactor and heated at 100 °C for 84 h to obtain white crystals. The white crystals were filtered and dried in an oven at 80 °C for 24 h. Then, 2.4 g of white crystals were dispersed in a mixture solution containing 50 mL methanol and 50 mL DMF. After that, the solution was added to a mixture solution containing 3.3 g of 2-aminoterephthalic acid and 75 mL of DMF. The final mixture was heated in an oven at 100 °C for 24 h. The resulting suspension was centrifuged at 7800 rpm for 5 min. Afterwards, the NH_2_-MIL-125 (Ti) fillers were washed several times with methanol and DMF before being dried in an oven at 60 °C for 24 h.

### 2.4. Fabrication of Dense Membrane

In our previous work [3], the 7.0 wt% NH_2_-MIL-125 (Ti) /6FDA-durene mixed matrix membrane showed the highest performance in terms of CO_2_ and CH_4_ single gas permeation; therefore, this membrane was utilized to obtain the optimum condition in CO_2_/CH_4_ binary gas separation. The mixed matrix membrane was prepared by dispersing fillers and dissolving the polymer in DCM separately. After stirring, the suspension was sonicated to disperse the fillers in DCM. Then, the priming step was introduced by adding 10 wt% of the polymer solution into the filler suspension under stirring to induce the polymer–filler interface [22] before the suspension was sonicated again. The remaining polymer solution was added to the suspension, which was then sonicated. Subsequently, the suspension was vigorously stirred for 1 h before being cast onto a petri dish. The petri dish was then covered with perforated aluminum foil for solvent evaporation at room temperature for 24 h. After complete evaporation, the membrane film was carefully removed from the petri dish and dried in an oven at 60 °C. Then, the membrane was further dried under vacuum at 60 °C for 24 h, followed by thermal annealing at 250 °C for 24 h.

### 2.5. Binary Gas Separation Measurement

Binary gas separation testing was carried out by using a custom-made permeation test rig as described in our previous work [3]. The compositions of gases in the retentate and permeate gas streams were determined using a gas analyzer (Fuji, NDIR Gas Analyzer ZPAJ). Then, the permeability of the gases was calculated using Equations (1) and (2), as follows [20]:

(1) $$P_{CO2}=\frac{V_{P}y_{CO2}t}{A_{m}\left(p_{h}x_{CO2}-p_{l}y_{CO2}\right)}$$

(2) $$P_{CH4}=\frac{V_{P}y_{CH4}t}{A_{m}\left(p_{h}x_{CH4}-p_{l}y_{CH4}\right)}$$

where $P_{CO2}$ and $P_{CH4}$ are the permeability of CO_2_ and CH_4_ (Barrer, 1 Barrer = 1 × 10^−10^ cm^3^(STP).cm/s·cm^2^·cmHg), *V*_p_ is the volumetric flowrate (cm^3^(STP)/s), *A*_m_ and *t* are the operational area (cm^2^) and the thickness (cm) of the mixed matrix membrane, respectively, *p_h_* and *p*_l_ are the pressure of feed and permeate side, respectively (cmHg), and *x* and *y* represent the volume fraction of the component in retentate and permeate streams by assuming ideal gas behaviors. The CO_2_/CH_4_ separation factor was calculated using Equation (3), as follows [20]:

(3) $$\alpha_{CO_{2}/CH_{4}}=\frac{y_{CO_{2}}/y_{CH_{4}}}{x_{CO_{2}}/x_{CH_{4}}}$$

where $\alpha_{CO2/CH4}\;$ indicates the *CO*_2_/*CH*_4_ separation factor, and *x* and *y* represent the volume fraction of the component in the retentate and permeate streams, respectively.

### 2.6. Optimization of Membrane Separation Performance

The optimization of CO_2_ separation from CH_4_ was conducted based on the experimental runs generated by Design-Expert software V9.0 (Stat-Ease Inc., Minneapolis, MN, USA). The central composite design (CCD) available in the software was selected due to its various advantages, including its flexibility, efficiency, and continuous run. In the current study, three operational parameters were selected, namely, feed pressure (A), temperature (B), and CO_2_ feed concentration (C). Meanwhile, three significant responses were determined, i.e., CO_2_ permeability, CH_4_ permeability and CO_2_/CH_4_ separation factor. All parameters were tested at three measurement levels from low to high range. Then, the alpha (α) was fixed at 1, which is considered to be a face-centered design. The range and level of the operational parameters are shown in Table 1.

The interaction between the operational parameters, i.e., feed pressure, temperature and CO_2_ feed concentration, and responses, i.e., the permeability of the gases and the CO_2_/CH_4_ separation factor, were further analyzed via analysis of variance (ANOVA). The experimental results were fitted into empirical models (second-order polynomial function) expressed in Equation (4) in order to correlate the corresponding responses over the operational parameters [18].

(4) $$Y=B_{0}+\sum_{i=1}^{3}B_{i}x_{i}+\sum_{i=1}^{2}\sum_{j=i+1}^{3}B_{ij}x_{i}x_{j}+\sum_{i=1}^{3}B_{ii}x_{i}{}^{2}$$

where *Y* is the corresponding response, *B*_0_ is a fixed term, and *B_i_*, *B_ij_* and *B_ii_* are linear, interaction and quadratic terms, respectively. Meanwhile, *x_i_* and *x_j_* represent the coded terms for operational parameters.

The fitted model for each corresponding response was analyzed in terms of the statistical significance of the operational parameters and interactions using the F- and *p*-values. The F-value is defined as the ratio of the mean square model to the mean square error. The mean square model is the sum of squares divided by the number of degrees of freedom. The *p*-value indicates the significance of the model; therefore, *p* < 0.05 shows that the regression model is relevant, and the null hypothesis (*H*_o_): *p* ≤ 0.5 is rejected. In addition, the consistency of the models was quantified by the coefficient of determination (R^2^). Residual is the unexplained variation by the fitted model. ‘Lack of fit’ denotes that the lack of fit is insignificant in comparison to the pure error.

## 3. Results and Discussion

### 3.1. Characteristics of Membrane

The physicochemical properties, morphology and filler distribution, thermal stability, fractional free volume (FFV) analysis as well as CO_2_ and CH_4_ single gas performance of the NH_2_-MIL-125 (Ti)/6FDA-durene membranes at various filler loadings were discussed in detail in our previous work [3]. As noted in our previous work, the 7.0 wt% NH_2_-MIL-125 (Ti)/6FDA-durene mixed matrix membrane exhibited the highest CO_2_ permeability and CO_2_/CH_4_ gas pair selectivity, i.e., 1115.7 Barrer and 37.1, respectively, at a feed pressure of 3.5 bar and at room temperature. In this work, the performance of this membrane in mixed gas separation with various operating parameters, particularly at high CO_2_ feed concentration, was investigated using a statistical approach consisting of a central composite design (CCD) paired with a response surface methodology (RSM). Figure S1 and Table S1 display the characterization results comprising the XRD, FESEM, EDX and FFV for the 7.0 wt% NH_2_-MIL-125 (Ti)/6FDA-durene mixed matrix membrane. In reference to Figure S1a, the significant peaks observed at 6.9° and 9.8° corresponded to the X-ray reflection planes (011) and (002) of the NH_2_-MIL-125 (Ti) fillers. Meanwhile, the fillers were encapsulated and uniformly dispersed in the 6FDA-durene polymer matrix, as shown in Figure S1b. On the other hand, Table S1 compares the CO_2_ and CH_4_ single gas permeation performance and FFV values of pure and 7.0 wt% NH_2_-MIL-125 (Ti)/6FDA-durene mixed matrix membrane.

### 3.2. Central Composite Design (CCD)

From CCD, 20 trial experiments, including eight factorial points, six axial points, and six central point replicates in a randomized sequence, were generated by the DoE software. The replicated center points were used to estimate the pure error for the lack of fit test. The randomized sequence for the experimental run was intended to limit the impact of uncontrollable factors. Therefore, experimental 20 runs were conducted and three significant responses, namely, CO_2_ permeability, CH_4_ permeability, and CO_2_/CH_4_ separation factor, were measured. Table 2 shows the condition of the operational parameters and significant responses of the membrane separation performance observed in the experiments. From Table 2, it can be observed that the permeability of CO_2_ and CH_4_ were in the range of 293.2 to 794.4 Barrer and 28.7 to 147.5 Barrer, respectively. The percentage errors between the actual and predicted values of membrane separation performances are listed in Table S2.

Meanwhile, the CO_2_/CH_4_ separation factor obtained ranged from 5.6 to 13.0. The margin of error of the experimental results was ±5%. Furthermore, compared to single gas permeation testing [3], the performance of the membrane in binary gas separation demonstrated lower values. These difference could be explained by the competitive sorption between CO_2_ and CH_4_ gas molecules [23]. The presence of a competitive gas may substantially affect the gas penetration over the membrane [24].

### 3.3. CO_2_ Permeability

The quadratic polynomial model suggested by the DoE software for CO_2_ permeability is shown in Equation (5) in the form of a coded value.

(5) $$\begin{array}{cc} CO_{2}Permeability_{coded}= & 518.31-67.77A+36.63B+98.10C-72.10AB-33.77AC+16.25BC+\\ & \;\;\;28.97A^{2}-117.49B^{2}+50.49C^{2} \end{array}$$

where *A*, *B* and *C* denote the feed pressure (bar), temperature (°C) and CO_2_ feed concentration (mol%), respectively.

Table 3 presents the findings of the ANOVA and regression analysis for CO_2_ permeability over the membrane. As shown in Table 3, model F and *p* values of 27.11 and <0.05 were achieved, respectively, suggesting that the model is statistically significant. In this case, *A*, *B*, *C*, *AB*, *AC*, *B*^2^, *C*^2^ are statistically significant model terms. It can be observed from Table 3 that an R^2^ value of 0.96 was achieved, which validated the accuracy of the model for CO_2_ permeability.

**Figure 1:**
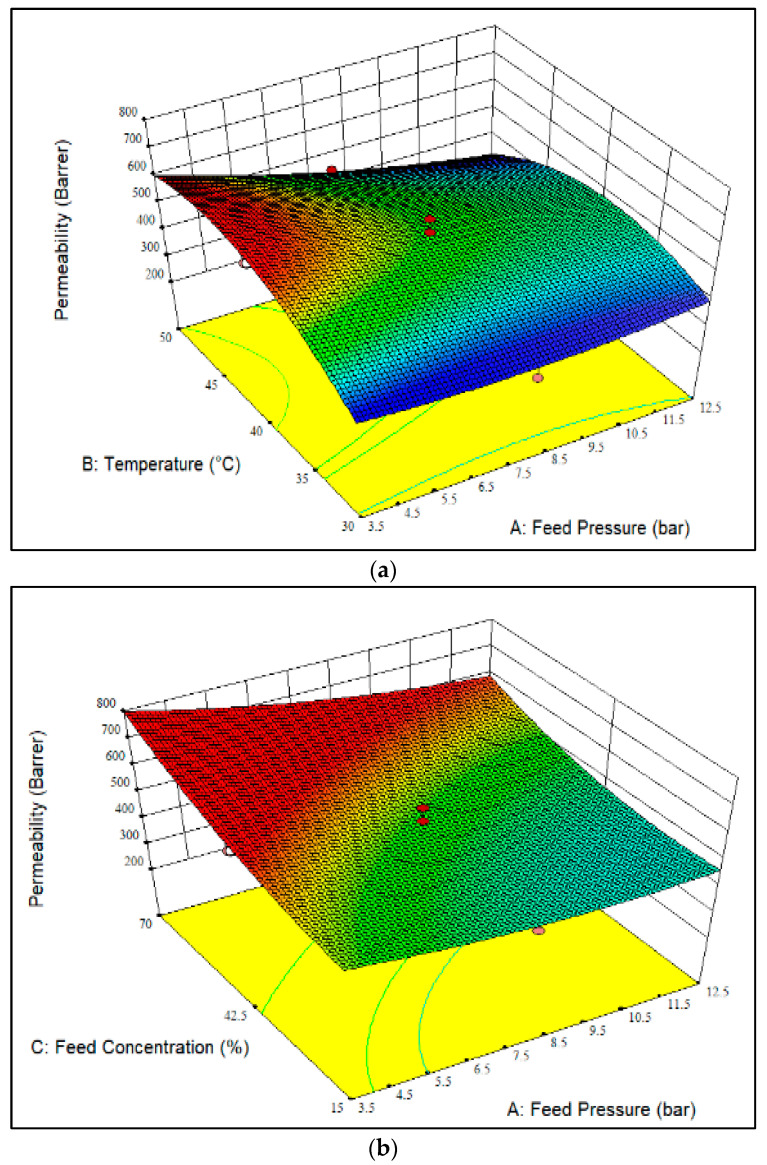

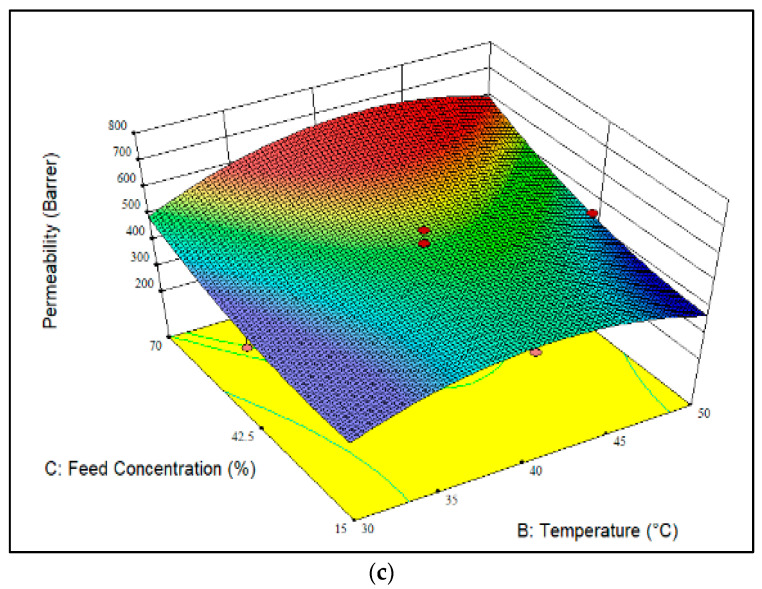
3D surface of the CO_2_ permeability as a function of feed pressure, CO_2_ feed concentration and temperature at (**a**) CO_2_ feed concentration of 42.5 mol%, (**b**) temperature of 40 °C and (**c**) feed pressure of 8 bar. (The darker colour (red) representing higher membrane performance whereas a lighter colour (blue) indicating lower membrane performance).

Figure 1a–c display a 3D plot of the effect of different operational parameters on CO_2_ permeability. The increment of temperature at a feed pressure of 3.5 bar and a fixed CO_2_ feed concentration of 42.5 mol% resulted in higher CO_2_ permeability, as observed in Figure 1a. This could be due to the higher CO_2_ diffusivity [19] caused by the increment of polymer chain mobility and free volume [18]. Meanwhile, the increment of feed pressure at temperatures between 30 to 40 °C resulted in a slight improvement in CO_2_ permeability due to the increase in gas solubility as a consequence of thermodynamic promotion [11]. On the other hand, a decreasing trend of CO_2_ permeability can be seen for temperatures ranging from 40 to 50 °C, mainly because of the CO_2_ sorption isotherm, which follows the dual-mode sorption mechanism [25]. Generally, higher temperatures lead to higher CO_2_ permeability; however, in this study, we observed a different trend. This might have been related to the lower solubility of gas molecules with increasing temperature. The adverse impact of temperature on sorption enthalpy would have affected the transport and sorption behavior of gases over the membrane [26]. From the obtained results, a maximum CO_2_ permeability of 609.1 Barrer was achieved at a feed pressure of 3.5 bar and a temperature of 40 °C. Meanwhile, the lowest CO_2_ permeability, i.e., 321.5 Barrer, was achieved at a feed pressure of 8 bar and a temperature of 30 °C.

Figure 1b shows the effect of CO_2_ feed concentration and feed pressure on CO_2_ permeability at a constant temperature of 40 °C. It can be seen in Figure 1b that the lowest CO_2_ permeability, i.e., 470.7 Barrer, was achieved at a CO_2_ feed concentration of 15 mol% and a feed pressure of 8 bar. In contrast, the highest CO_2_ permeability, 792.2 Barrer, was obtained at a feed pressure of 3.5 bar and a CO_2_ feed concentration of 70 mol%. Additionally, at constant temperature, increasing the CO_2_ feed concentration in the feed caused significant improvement in CO_2_ permeability; this was mainly due to the higher sorption of CO_2_ than CH_4_ over the membrane. Furthermore, the interaction between CO_2_ molecules and -NH_2_ elements also contributed to the improvement of CO_2_ diffusion through the membrane [27]. On the other hand, the CO_2_ permeability decreased slightly with increasing feed pressure. This result was attributed to the saturation of available sorption sites, which resulted in a lower solubility coefficient [28]. Furthermore, this trend was also related to the dual-mode sorption and diffusion mechanism on gas transport behavior over the membrane.

Figure 1c illustrates the effect of CO_2_ feed concentration and temperature on CO_2_ permeability at a fixed feed pressure of 8 bar. As shown in Figure 1c, increasing the CO_2_ feed concentration led to an increase in CO_2_ permeability due to the higher CO_2_ sorption effect relative to CH_4_, which, subsequently, enhanced the sorption of CO_2_ over CH_4_ gas molecules in the membrane. A similar incremental trend of CO_2_ permeability was observed under fixed feed pressure conditions, as shown in Figure 1b. When the temperature increased from 30 °C to 40 °C at a constant feed pressure of 8 bar, the CO_2_ permeability improved due to the increase in movement and flexibility of polymer chains, as well as the kinetic energy of gas molecules [26]. Furthermore, it could be that the impact of temperature on the gas transport and the sorption behavior compensated for the adverse effect of temperature on gas solubility. However, a slight decrease in CO_2_ permeability was discovered after increasing the temperature from 40 °C to 50 °C. This was mainly due to the sorption competition between the CO_2_ and CH_4_ gases [25]. The reduction in gas solubility caused by the reduced interaction of CO_2_ gas with the polymer matrix was attributed to the increment of adsorption energy with temperature; consequently, the thermodynamic effect overcame the kinetic effect during gas penetration [29]. From Figure 1c, it may be seen that the maximum CO_2_ permeability, i.e., 650.2 Barrer, was achieved at a feed pressure of 8 bar, a CO_2_ feed concentration of 70 mol% and a temperature of 40 °C. A parity plot for CO_2_ permeability is shown in Figure S2a. It can be seen that the actual and predicted values of the responses were scattered near to the 95% prediction limits.

### 3.4. CH_4_ Permeability

Equation (6) depicts the quadratic polynomial model for CH_4_ permeability in terms of coded value.

(6) $$\begin{array}{cc} CH_{4}Permeability_{coded}= & 55.48-23.89A+13.76B+10.52C-12.10AB-7.01AC+7.08BC-0.15A^{2}-\\ & \;\;\;\;\;\;24.03B^{2}+36.76C^{2} \end{array}$$

where *A*, *B* and *C* denote the feed pressure (bar), temperature (°C) and CO_2_ feed concentration (mol%), respectively.

Table 4 demonstrates the ANOVA and regression analysis of CH_4_ permeability. As shown, F and *p* values of 29.95 and < 0.05, respectively, were achieved, indicating that the model terms were statistically significant. Additionally, *A*, *B*, *C*, *AB*, *AC*, *BC*, *B*^2^ and *C*^2^ were statistically significant model terms in this context. In addition, an R^2^ value of 0.96 was obtained, confirming the accuracy of the model for CH_4_ permeability.

**Figure 2:**
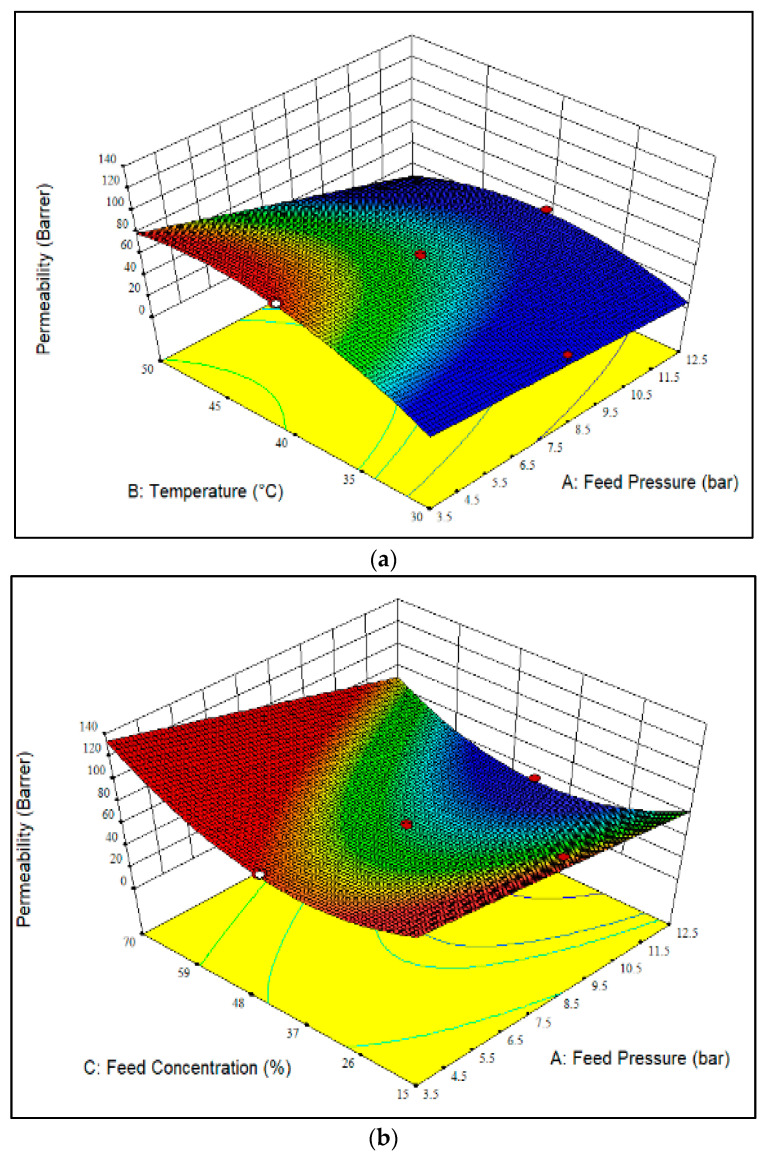

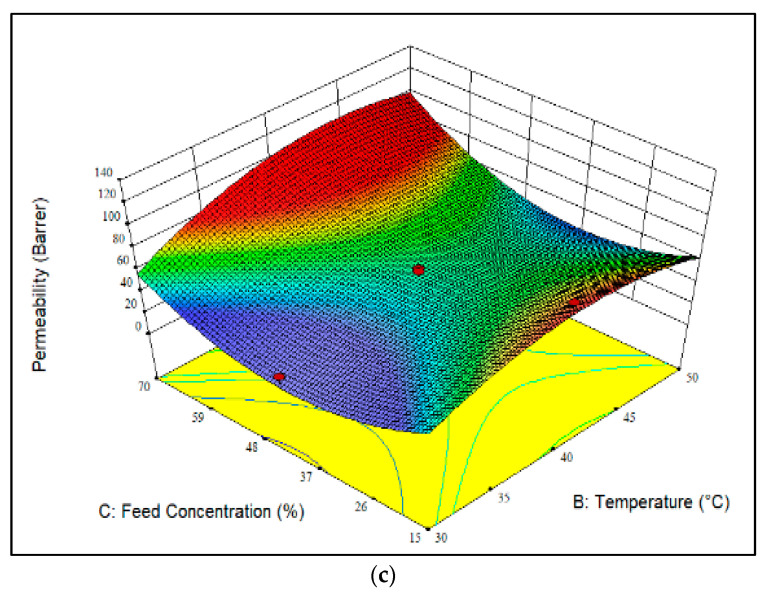
CH_4_ permeability as a function of feed pressure, CO_2_ feed concentration and temperature at (**a**) CO_2_ feed concentration of 42.5 mol%, (**b**) temperature of 40 °C and (**c**) feed pressure of 8 bar. (The darker color (red) represents higher membrane performance whereas the lighter color (blue) indicated lower membrane performance.)

Figure 2a–c display a 3D plot of the CH_4_ permeability over the membrane. The increment of temperature from 30 °C to 40 °C resulted in higher CH_4_ permeability, as observed in Figure 2a. The increase in chain mobility and free volume resulted in higher diffusivity, and thus, increased the penetration of CH_4_ molecules through the membrane [30]. The relationship between temperature and permeability can be defined using Arrhenius equation, where CH_4_ molecules exhibit higher activation energy than CO_2_ molecules, and therefore, promote the diffusion of nonpolar gases over the glassy polymer. Meanwhile, a reduction of CH_4_ permeability was found with the increment of temperature from 40 °C to 50 °C. This was ascribed to the reduction in solubility, which subsequently enhanced the restriction of CH_4_ gas permeation through the membrane [31]. Meanwhile, increasing the feed pressure caused a slight reduction in CH_4_ permeability, mainly due to the decrease in the dual-sorption properties for CH_4_ gas [19], as well as the high compressibility of CH_4_ gas molecules [32] in the membrane.

Figure 2b shows the effect of CO_2_ feed concentration and feed pressure on CH_4_ permeability at a constant temperature of 40 °C. Referring to Figure 2b, the CH_4_ permeability obtained at feed pressures ranging from 3.5 bar to 12.5 bar demonstrated a similar trend to the results presented in Figure 2a. On the other hand, an increase in CO_2_ feed concentration from 15 mol% to 42.5 mol% led to a reduction of CH_4_ permeability, due to the lower sorption of CH_4_ compared to CO_2_, as well as the slower diffusion of CH_4_ gas over the membrane [20]. However, increasing the CO_2_ feed concentration from 42.5 mol% to 70 mol% enhanced the permeability of CH_4_. The increasing trend in CH_4_ permeability was because of the swelling of polymer chain packing at higher CO_2_ feed concentrations, and therefore, increased the segmental mobility [19]. On the other hand, the lowest CH_4_ permeability, i.e., 33.9 Barrer, was seen with a CO_2_ feed concentration of 42.5 mol% and a feed pressure of 12.5 bar. In contrast, the maximum CH_4_ permeability, 133.3 Barrer, was achieved at a CO_2_ feed concentration of 70 mol%, a temperature of 40 °C and a feed pressure of 3.5 bar (Figure 2b).

Figure 2c demonstrates the effect of CO_2_ feed concentration and temperature on CH_4_ permeability at a constant feed pressure of 8 bar. As shown, the CH_4_ permeability gradually improved with increasing the temperature, owing to the enhanced mobility and flexibility of the polymer chains [26], as well as to the higher activation energy of CH_4_ [33]. Meanwhile, a reduction in CH_4_ permeability was observed at CO_2_ feed concentrations ranging from 15 mol% to 42.5 mol%, and an improvement in CH_4_ permeability was found when the CO_2_ feed concentration increased from 42.5 mol% to 70 mol%. This trend was identical to that shown in Figure 2b. Apart from that, the highest CH_4_ permeability, i.e., 106.1 Barrer, was obtained with a CO_2_ feed concentration of 70 mol%, a feed pressure of 8 bar and a temperature of 40 °C (Figure 2c); this was associated with concentration polarization effects [34]. Meanwhile, the lowest CH_4_ permeability, 28.7 Barrer, was observed at a CO_2_ feed concentration of 42.5 mol% and a temperature of 30 °C. The increase in CH_4_ permeability with the increase in temperature and CO_2_ feed concentration was mainly due to the increase in polymer free volume caused by the alteration of polymer chain packing and intersegmental motion [33], as well as the swelling effect induced by CO_2_ gas. The presence of high CO_2_ feed concentrations caused the rapid diffusion of CH_4_ gas through the membrane [35], because CH_4_ gas molecules are more accessible to a swollen polymer matrix. A parity plot for CH_4_ permeability is shown in Figure S2b. As shown, very few data points fall outside the 95% prediction limits.

### 3.5. CO_2_/CH_4_ Separation Factor

The quadratic polynomial model for CO_2_/CH_4_ separation factor is defined in Equation (7) in the form of coded values.

(7) $$\begin{array}{cc} Separation\;Factor_{coded}= & 9.93+1.82A-1.05B+0.84C-0.54AB+0.46AC-0.80BC+0.30A^{2}+\\ & \;\;\;\;\;0.11B^{2}-2.70C^{2} \end{array}$$

where *A*, *B* and *C* denote the feed pressure (bar), temperature (°C) and CO_2_ feed concentration (mol%), respectively.

The ANOVA analysis of the CO_2_/CH_4_ separation factor is presented in Table 5. It can be seen that model F and *p* values of 18.04 and <0.05 were achieved, respectively, indicating that the model terms are statistically significant, while *A*, *B*, *C*, *BC* and *C*^2^ are statistically significant model terms. Furthermore, an R^2^ value of 0.94 was achieved, showing the accuracy of the model for CO_2_/CH_4_ separation factor.

**Figure 3:**
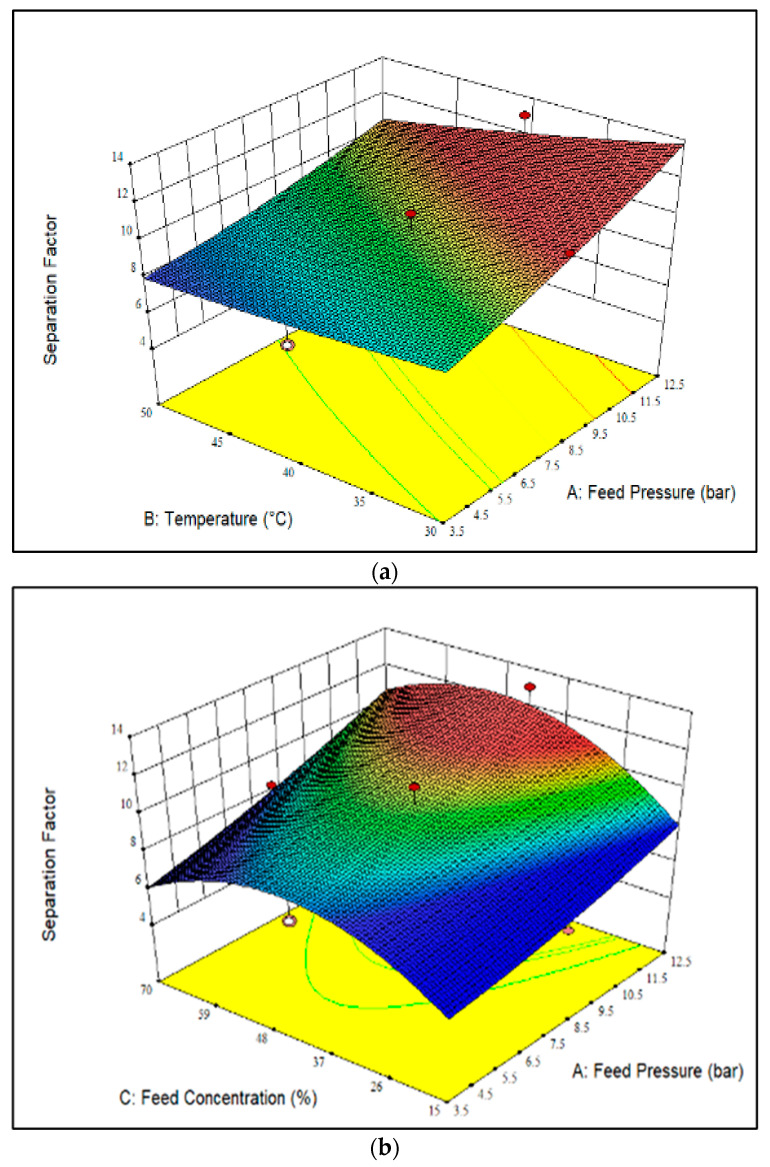

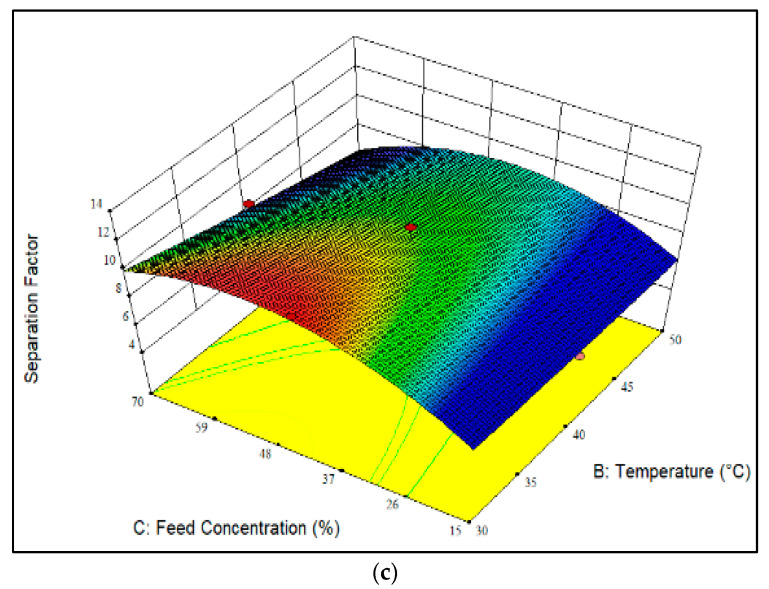
3D surface of the CO_2_/CH_4_ separation factor as a function of feed pressure, CO_2_ feed concentration and temperature at (**a**) CO_2_ feed concentration of 42.5 mol%, (**b**) temperature of 40 °C and (**c**) feed pressure of 8 bar. (The darker colour (red) represents higher membrane performance whereas the lighter color (blue) indicates lower membrane performance.).

Figure 3 displays 3D plots of the effect of feed pressure, temperature, and CO_2_ feed concentration on the CO_2_/CH_4_ separation factor over the membrane. As shown, increasing the feed pressure resulted in a higher CO_2_/CH_4_ separation factor owing to the higher CO_2_ adsorption capacity compared to CH_4_, as well as the good compatibility of the polymer-filler, which was induced by the presence of -NH_2_ groups in the membrane [27]. The enhanced CO_2_/CH_4_ separation factor was related to the improvement of CO_2_ permeability and the reduction of CH_4_ permeability observed in Figure 1a and Figure 2a. In contrast, increasing the temperature revealed a negligible impact on CO_2_/CH_4_ separation factor, since the contribution of solubility and diffusivity is interchangeable. Additionally, as shown in Figure 3a, the highest CO_2_/CH_4_ separation factor, i.e., 13.0, was obtained at a temperature of 30 °C and a feed pressure of 12.5 bar. In comparison, the lowest CO_2_/CH_4_ separation factor, 7.3, was observed at a feed temperature of 50 °C and a feed pressure of 3.5 bar.

Figure 3b illustrates the effect of CO_2_ feed concentration and feed pressure on the CO_2_/CH_4_ separation factor at a constant temperature of 40 °C. It can be observed that the CO_2_/CH_4_ separation factor increased with increasing feed pressure. This was mainly due to the higher sorption and permeation of CO_2_ molecules over the membrane compared to CH_4_ [33], which resulted in higher CO_2_ permeability and lower CH_4_ permeability, as observed in Figure 1b and Figure 2b, respectively. The higher CO_2_ permeability also contributed to the increase in CO_2_ solubility and the reduction of CH_4_ solubility, and thus, improved the CO_2_/CH_4_ solubility selectivity [36]. Furthermore, the CO_2_/CH_4_ separation factor displayed an increasing trend with increasing CO_2_ feed concentration up to 42.5 mol%, and then decreased when the CO_2_ feed concentration was increased further to 70 mol%. The increment of CO_2_ feed concentration caused a reduction of CH_4_ permeability and led to an increase in CO_2_/CH_4_ separation factor, owing to the smaller kinetic diameter of CO_2_ compared to CH_4_. Meanwhile, increasing the CO_2_ feed concentration further from 42.5 mol% to 70 mol% caused an improvement of CH_4_ permeability, but a reduction in the CO_2_/CH_4_ separation factor was observed due to the early stage of CO_2_-induced plasticization behavior [33,37]. The highest separation factor, i.e., 13.0, was achieved at a feed pressure of 12.5 bar and CO_2_ a feed concentration of 42.5 mol%. Meanwhile, the lowest CO_2_/CH_4_ separation factor, 6.0, was observed at a feed pressure of 3.5 bar and a CO_2_ feed concentration of 15 mol%, mainly resulting from the presence of a larger amount of CH_4_ gas (85 mol%) in the feed mixture.

Figure 3c shows the effect of CO_2_ feed concentration and temperature on the CO_2_/CH_4_ separation factor at a pressure of 8 bar. From Figure 3c, increasing the temperature at a fixed feed pressure of 8 bar caused a slightly drop in the CO_2_/CH_4_ separation factor. This reduction was mainly due to the improvement of CO_2_ and CH_4_ permeability, as explained in Section 3.3 and Section 3.4. Furthermore, at a fixed feed pressure of 8 bar, the increase of CO_2_ feed concentration from 15 mol% to 42.5 mol% resulted in the enhancement of the CO_2_/CH_4_ separation factor, which was consistent with the increase of CO_2_ permeability, as shown in Figure 1c. On the other hand, increasing the CO_2_ feed concentration from 42.5 mol% to 70 mol% led to a slight drop in the CO_2_/CH_4_ separation factor due to the saturation of CO_2_ gas molecules inside the polymer voids [11]. A parity plot for CO_2_/CH_4_ separation factor is shown in Figure S2c. Overall, the actual and predicted values of the responses were scattered near the 95% prediction limits. As shown in Figure S2c, very few data points fall outside the 95% prediction limits.

### 3.6. Optimization of CO_2_/CH_4_ Separation Performance

The primary objective of this work is to identify the optimal operational conditions for membrane separation in terms of CO_2_ permeability and CO_2_/CH_4_ separation factor. Therefore, the optimization conditions included maximizing the CO_2_ feed concentration as an operational parameter, as well as CO_2_ permeability and CO_2_/CH_4_ separation factor. Additionally, the desirability function (DF) was used in RSM to optimize a sequence of quadratic models. The geometric mean of individual desirability (d) was used to calculate the total desirability (D), as expressed in Equation (8) [38]:

(8) Dd=(d1×d2×…dn)1n

where *D*_d_ is the total desirability and *d*_n_ is the nth desirability, *n* = 1, 2, …, n. The total desirability was measured from 0 to 1, where 0 represents the most undesirable response and 1 the most desirable.

The optimum solution and its desirability generated by the DOE software is listed in Table 6 in term of actual values. The optimum operational parameters suggested for CO_2_ feed concentration, feed pressure, and temperature were 70 mol%, 12.5 bar and 34.7 °C, respectively, which yielded optimal CO_2_ permeability and separation factor values, i.e., 571.9 Barrer and 11.9, respectively.

### 3.7. Validation of the Optimum Condition

In order to validate the optimum conditions and predicted responses shown in Table 6, three repeated experiments were conducted based on the suggested conditions; the results are presented in Table 7. The percentage (%) error for CO_2_ permeability ranged from 3.6% to 6.5% relative to the standard deviation of 1.5%. On the other hand, the percentage (%) error for CO_2_/CH_4_ separation factor ranged from 0.8% to 5.0% with a standard deviation of 2.1%. Meanwhile, the average errors obtained for CO_2_ permeability and CO_2_/CH_4_ separation factor were 5.3% and 2.8%, respectively. Overall, the average error for the experimental and predicted values were within 5%, indicating that the model validity reached 95% of the prediction interval. Consequently, the model was successfully validated, and as such, the optimization of operational parameters was achieved utilizing the RSM approach.

## 4. Conclusions

In conclusion, the effects of operational parameters, i.e., feed pressure, temperature and CO_2_ feed concentration, were identified as dominant factors influencing the separation performance of a NH_2_-MIL-125 (Ti)-6FDA/durene membrane. CO_2_ feed concentration demonstrated a significant effect on CO_2_ permeability, whereas feed pressure was the primary parameter influencing CH_4_ permeability and CO_2_/CH_4_ separation factor, with F-values of 102.5 and 59.3, respectively. The R^2^ values obtained ranged from 0.94 to 0.96, indicating that the regression models were statistically significant. The optimum operational parameters, i.e., a feed pressure of 12.5 bar, a temperature of 34.7 °C and a CO_2_ feed concentration of 70 mol%, yielded the maximum CO_2_ permeability, i.e., 609.3 Barrer, and a CO_2_/CH_4_ separation factor of 11.6. The average errors for CO_2_ permeability and CO_2_/CH_4_ separation factor were 5.3% and 2.8%, respectively, suggesting that the model was 95% reliable. Overall, the experimental findings show that the RSM paired with the CCD method is a better strategy to obtain optimal CO_2_/CH_4_ separation performance with a NH_2_-MIL-125 (Ti)/6FDA-based mixed matrix membrane. The present research provides an experimental reference for further improvements and scale-ups of membranes in CO_2_/CH_4_ separation with high CO_2_ feed concentrations.

## Figures and Tables

**Table 1:** Experimental range and levels of the operational parameters.

Operational Parameters	Units	Coded Measurement Levels		
		−1 (Low)	0 (Center)	1 (High)
Feed Pressure (A)	Bar	3.5	8.0	12.5
Temperature (B)	°C	30.0	40.0	50.0
CO_2_ Feed Concentration (C)	(mol%)	15.0	42.5	70.0

**Table 2:** CCD design matrix of 2^3^ factorial generated by DOE and the obtained significant responses.

Run	Operational Parameters			Significant Responses		
	A: Pressure (Bar)	B: Temperature (°C)	C: CO_2_ Concentration (mol%)	CO_2_ Permeability (Barrer)	CH_4_ Permeability (Barrer)	CO_2_/CH_4_ Separation Factor
1	3.5	40.0	42.5	569.6	58.3	9.9
2	8.0	40.0	42.5	569.6	58.3	9.9
3	3.5	50.0	70.0	326.2	52.3	5.5
4	12.5	30.0	70.0	609.1	81.9	7.3
5	8.0	40.0	70.0	450.2	92.7	6.0
6	8.0	50.0	42.5	504.0	39.9	12.8
7	3.5	30.0	70.0	448.2	33.9	13.0
8	12.5	50.0	70.0	293.2	36.2	7.3
9	12.5	30.0	15.0	506.6	83.2	6.1
10	8.0	40.0	15.0	419.7	39.7	8.6
11	12.5	40.0	42.5	794.4	147.5	5.3
12	8.0	40.0	42.5	442.9	39.5	8.6
13	3.5	50.0	15.0	502.3	47.4	10.8
14	8.0	40.0	42.5	321.5	28.7	11.4
15	3.5	30.0	15.0	464.4	58.4	8.1
16	8.0	30.0	42.5	650.2	97.0	8.4
17	8.0	40.0	42.5	520.3	55.5	9.3
18	12.5	50.0	15.0	567.5	74.3	7.5
19	8.0	40.0	42.5	520.3	55.5	9.3
20	8.0	40.0	42.5	510.1	91.0	6.1

**Table 3:** Analysis of variance (ANOVA) for CO_2_ permeability.

Source	Sum of Squares	Degree of Freedom	Mean Square	F-Value	Prob > F
Model	2.471 × 10^5^	9	27,456.76	27.11	<0.0001 ^a^
A-Pressure	45,922.31	1	45,922.31	45.34	<0.0001 ^a^
B-Temperature	13,418.30	1	13,418.30	13.25	0.0045 ^a^
C-Concentration	96,228.25	1	96,228.25	95.00	<0.0001 ^a^
AB	41,584.40	1	41,584.40	41.05	<0.0001 ^a^
AC	9123.30	1	9123.30	9.01	0.0133 ^a^
BC	2111.85	1	2111.85	2.08	0.1794 ^b^
A^2^	2307.53	1	2307.53	2.28	0.1621 ^b^
B^2^	37,959.26	1	37,959.26	37.47	0.0001 ^a^
C^2^	7009.65	1	7009.65	6.92	0.0251 ^a^
Residual	10,129.32	10	1012.93		
Lack of Fit	5282.07	5	1056.41	1.09	0.4636 ^b^
R^2^	0.96				

^a^ statistically significant, ^b^ statistically not significant.

**Table 4:** Analysis of variance (ANOVA) for CH_4_ permeability.

Source	Sum of Squares	Degree of Freedom	Mean Square	F-Value	Prob > F
Model	15,005.47	9	1667.27	29.95	<0.0001 ^a^
A-Pressure	5708.28	1	5708.28	102.54	<0.0001 ^a^
B-Temperature	1892.55	1	1892.55	34.00	0.0002 ^a^
C-Concentration	1106.91	1	1106.91	19.88	0.0012 ^a^
AB	1171.28	1	1171.28	21.04	0.0010 ^a^
AC	393.68	1	393.68	7.07	0.0239 ^a^
BC	400.73	1	400.73	7.20	0.0230 ^a^
A^2^	0.066	1	0.066	1.180 × 10^−3^	0.9733 ^b^
B^2^	1587.90	1	1587.90	28.52	0.0003 ^a^
C^2^	3716.16	1	3716.16	66.75	<0.0001 ^a^
Residual	556.69	10	55.67		
Lack of Fit	429.72	5	85.94	3.38	0.1035 ^b^
R^2^	0.96				

^a^ statistically significant, ^b^ statistically not significant.

**Table 5:** Analysis of variance (ANOVA) for CO_2_/CH_4_ separation factor.

Source	Sum of Squares	Degree of Freedom	Mean Square	F-Value	Prob > F
Model	91.12	9	10.12	18.04	<0.0001 ^a^
A-Pressure	33.27	1	33.27	59.29	<0.0001 ^a^
B-Temperature	10.96	1	10.96	19.54	0.0013 ^a^
C-Concentration	7.12	1	7.12	12.69	0.0052 ^a^
AB	2.38	1	2.38	4.23	0.0666 ^b^
AC	1.71	1	1.71	3.05	0.1113 ^b^
BC	5.18	1	5.18	9.24	0.0125 ^a^
A^2^	0.25	1	0.25	0.44	0.5229 ^b^
B^2^	0.036	1	0.036	0.064	0.8057 ^b^
C^2^	20.06	1	20.06	35.75	0.0001 ^a^
Residual	5.61	10	0.56		
Lack of Fit	3.43	5	0.69	1.57	0.3168 ^b^
R^2^	0.94				

^a^ statistically significant, ^b^ statistically not significant.

**Table 6:** Experimental conditions generated by the DOE software and responses.

Pressure (bar)	Temperature (°C)	Concentration (mol%)	CO_2_ Permeability (Barrer)	CH_4_ Permeability (Barrer)	CO_2_/CH_4_ Separation Factor	Desirability
12.5	34.7	70.0	571.9	60.4	11.9	0.8

**Table 7:** Validation of optimal condition for membrane separation.

Run	CO_2_ Permeability			Separation Factor		
	Actual (Barrer)	Predicted (Barrer)	Error (%)	Actual	Predicted	Error (%)
1	609.3	571.9	6.5	11.6	11.9	2.5
2	592.7	571.9	3.6	11.8	11.9	0.8
3	605.3	571.9	5.8	11.3	11.9	5.0
Average (%)			5.3	Average (%)		2.8
Standard deviation			1.5	Standard deviation		2.1

## Data Availability

The data presented in this study are available on request from the corresponding author.

## Supplementary Materials

The following supporting information can be downloaded at https://www.mdpi.com/article/10.3390/polym14071371/s1; Figure S1: The structural properties of the 7.0 wt NH_2_-MIL-125 (Ti)/6FDA-durene membrane including XRD, FESEM and EDX; Table S1: The FFV values and single gas permeation performance of membranes; Figure S2: The parity plot of predicted and actual data for the model of (a) CO_2_ permeability, (b) CH_4_ permeability, and (c) CO_2_/CH_4_ separation factor with a 95% prediction interval.; Table S2: The actual and predicted values of the membrane separation performances with percentage of error.
