# Supplementary material for: RSM Modeling and Optimization of CO2 Separation from High CO2 Feed Concentration over Functionalized Membrane
Source: Polymers (Basel). 2022 Mar 28;14(7):1371. doi: 10.3390/polym14071371 (PMC9003211; doi:10.3390/polym14071371)
Supplement: Supplementary file 1 [file polymers-14-01371-s001.zip › polymers-1614737-supplementary.pdf]

Supplementary File

Article

RSM Modeling and Optimization of CO_2 Separation from High CO_2 Feed Concentration over Functionalized Membrane

Nadia Hartini Suhaimi [1,2], Yin Fong Yeong [1,2,*], Norwahyu Jusoh [1,2], Thiam Leng Chew [1,2], Mohammad Azmi Bustam [1,3] and Muhammad Mubashir [4]

[1] Department of Chemical Engineering, Universiti Teknologi PETRONAS, Bandar Seri Iskandar 32610,
Malaysia; nadia_17005943@utp.edu.my (N.H.S.); norwahyu.jusoh@utp.edu.my (N.J.); thiamleng.chew@utp.edu.my (T.L.C.); azmibustam@utp.edu.my (M.A.B.)

[2] CO_2 Research Centre (CO_2RES), R&D Building, Universiti Teknologi PETRONAS, Bandar Seri Iskandar 32610, Malaysia

[3] Centre of Research in Ionic Liquids (CORIL), Universiti Teknologi PETRONAS, Bandar Seri Iskandar 32610, Malaysia

[4] Department of Petroleum Engineering, Faculty of Computing, Engineering & Technology, School of Engineering, Asia Pacific University of Technology, and Innovation, Kuala Lumpur 57000, Malaysia;
mubashir@apu.edu.my

* Correspondence: yinfong.yeong@utp.edu.my

Contents

1. Supplementary Tables S1 and S2
2. Supplementary Figures S1 and S2

Table S1. The FFV values and single gas permeation performance of membranes.

**Membrane**	**CO_2 Permeability (Barrer)**	**CH_4 Permeability (Barrer)**	**CO_2/CH_4 Separation Factor**	**Fractional Free Volume (FFV)**	**Ref.**
6FDA-durene	510.3	59.3	8.6	0.1981	[1]
7.0 wt NH_2-MIL-125 (Ti)/6FDA-durene	1115.7	30.1	37.1	0.2665	[1]

References:

1. Suhaimi, N. H., et al. (2020). "Separation of CO_2 from CH_4 using mixed matrix membranes incorporated with amine functionalized MIL-125 (Ti) nanofiller." Chemical Engineering Research and Design 159: 236-247.

(a)

(b)

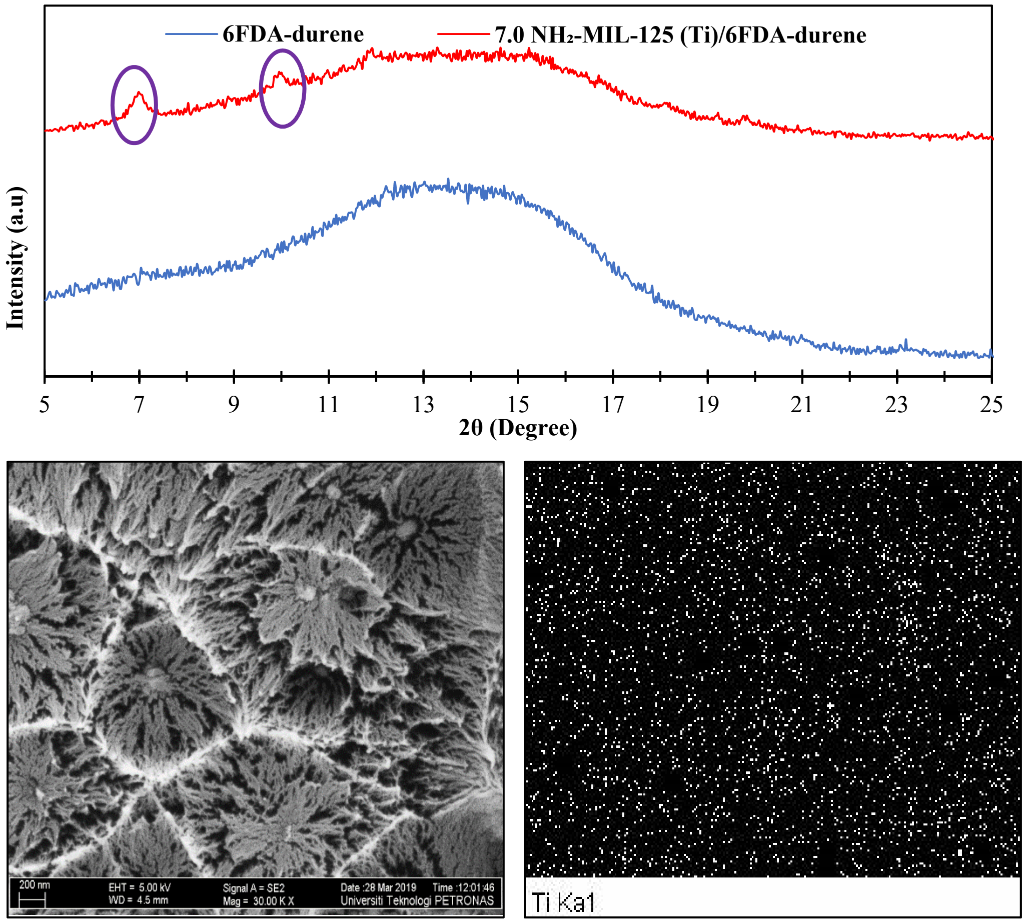

Figure S1. The structural properties of the 7.0 wt NH_2-MIL-125 (Ti)/6FDA-durene membrane including XRD (**a**), FESEM and EDX (**b**) [1].

References:

1. Suhaimi, N. H., et al. (2020). "Separation of CO_2 from CH_4 using mixed matrix membranes incorporated with amine functionalized MIL-125 (Ti) nanofiller." Chemical Engineering Research and Design 159: 236-247.

Table S2. The actual and predicted values of the membrane separation performances with percentage of error.

Run	CO_2 Permeability (Barrer)			CH_4 Permeability (Barrer)			CO_2/CH_4 Separation Factor		
	Actual	Predicted	Error (%)	Actual	Predicted	Error (%)	Actual	Predicted	Error (%)
1	569.55	518.81	9.78	58.26	59.71	2.43	9.85	9.37	5.07
2	569.55	518.81	9.78	58.26	59.71	2.43	9.85	9.37	5.07
3	326.19	323.62	0.80	52.33	54.99	4.83	5.49	5.22	5.18
4	609.06	615.35	1.02	81.94	81.82	0.14	7.29	8.06	9.59
5	450.15	471.01	4.43	92.68	84.32	9.91	5.95	6.04	1.55
6	503.91	495.98	1.60	39.86	38.29	4.10	12.83	13.26	3.21
7	448.24	479.82	6.58	33.94	34.04	0.29	13.01	11.71	11.09
8	293.24	296.39	1.06	36.16	34.59	4.53	7.39	7.46	0.92
9	506.56	518.81	2.36	83.20	59.71	39.34	6.08	9.37	35.15
10	419.72	399.82	4.98	39.74	45.43	12.53	8.63	9.03	4.46
11	794.37	804.80	1.30	147.46	141.77	4.01	5.25	4.98	5.45
12	442.92	437.76	1.18	39.48	47.81	17.43	8.56	8.66	1.10
13	502.30	518.81	3.18	47.43	59.71	20.57	10.78	9.37	14.99
14	321.47	364.50	11.80	28.65	20.30	41.14	11.37	10.75	5.77
15	464.43	457.54	1.51	58.41	55.76	4.76	8.06	8.46	4.75
16	650.19	667.20	2.55	97.03	105.37	7.91	8.35	7.73	8.00
17	520.32	518.81	0.29	55.54	59.71	6.99	9.32	9.37	0.59
18	567.47	554.85	2.27	74.33	75.90	2.07	7.53	7.59	0.82
19	520.32	518.81	0.29	55.54	59.71	6.99	9.32	9.37	0.59
20	510.11	508.58	0.30	90.97	92.55	1.70	6.12	5.83	5.05

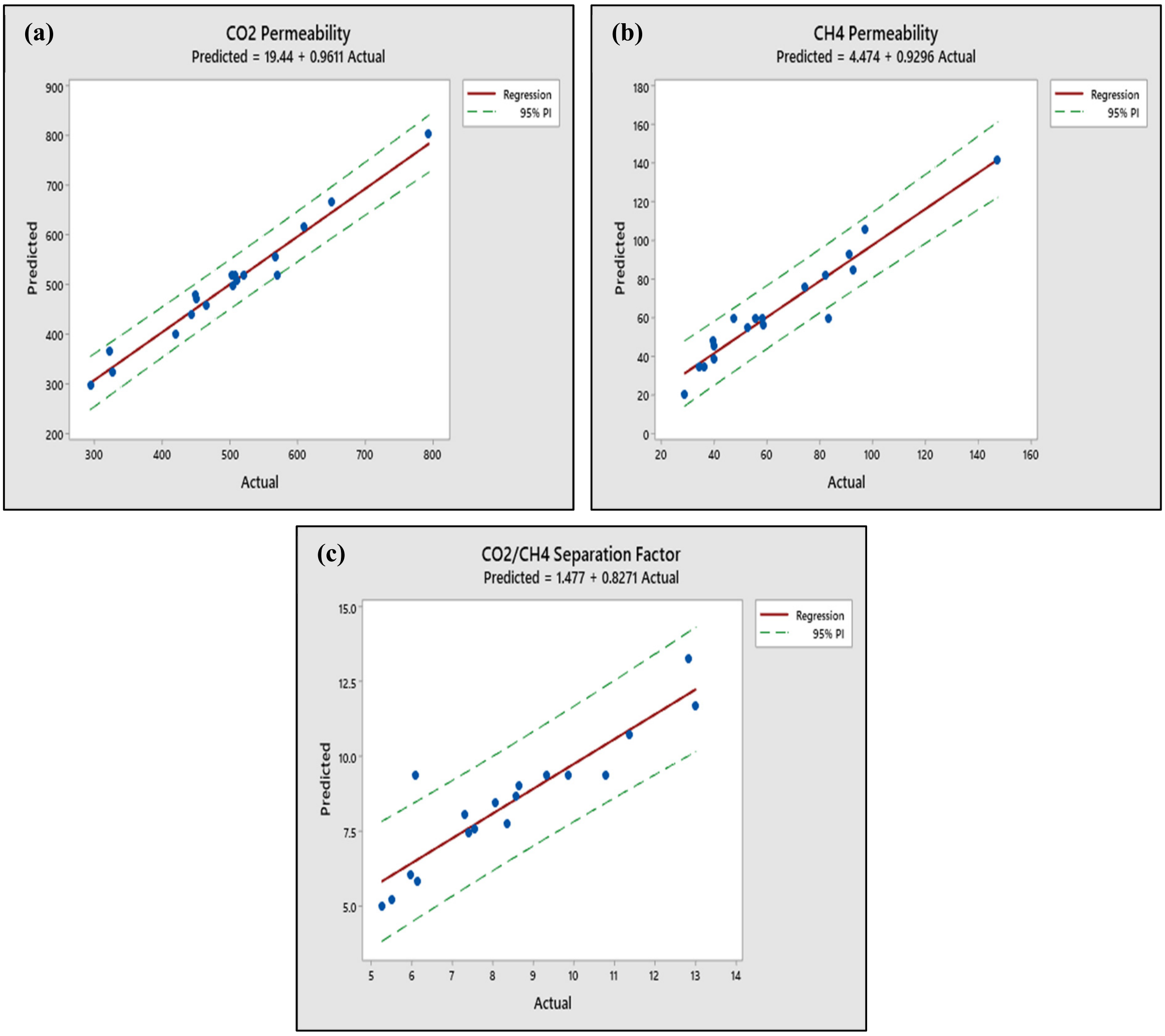

Figure S2. The parity plot of predicted and actual data for the model of (**a**) CO_2 permeability, (**b**) CH_4 permeability, and (**c**) CO_2/CH_4 separation factor with a 95% prediction interval.
